# Response Characterization of an Inexpensive Aerosol Sensor

**DOI:** 10.3390/s17122915

**Published:** 2017-12-15

**Authors:** Joel Kuula, Timo Mäkelä, Risto Hillamo, Hilkka Timonen

**Affiliations:** Finnish Meteorological Institue, Erik Palmenin aukio 1, FIN-00560 Helsinki, Finland; timo.makela@fmi.fi (T.M.); risto.hillamo@fmi.fi (R.H.); hilkka.timonen@fmi.fi (H.T.)

**Keywords:** inexpensive aerosol sensor, novel evaluation method, particulate matter

## Abstract

Inexpensive aerosol sensors have been considered as a complementary option to address the issue of expensive but low spatial coverage air quality monitoring networks. However, the accuracy and response characteristics of these sensors is poorly documented. In this study, inexpensive Shinyei PPD42NS and PPD60PV sensors were evaluated using a novel laboratory evaluation method. A continuously changing monodisperse size distribution of particles was generated using a Vibrating Orifice Aerosol Generator. Furthermore, the laboratory results were validated in a field experiment. The laboratory tests showed that both of the sensors responded to particulate mass (PM) concentration stimulus, rather than number concentration. The highest detection efficiency for the PPD42NS was within particle size range of 2.5–4 µm, and the respective optimal size range for the PPD60PV was 0.7–1 µm. The field test yielded high PM correlations (R^2^ = 0.962 and R^2^ = 0.986) for viable detection ranges of 1.6–5 and 0.3–1.6 µm, when compared to a medium cost optical dust monitor. As the size distribution of atmospheric particles tends to be bimodal, it is likely that indicatively valid results could be obtained for the PM_10–2.5_ size fraction (particulate mass in size range 2.5–10 µm) with the PPD42NS sensor. Respectively, the PPD60PV could possibly be used to measure the PM_2.5_ size fraction (particulate mass in size below 2.5 µm).

## 1. Introduction

Mapping of spatial distribution of particulate matter (PM) concentrations requires high resolution monitoring networks. Currently, high unit cost and complexity of the standardized continuous monitoring instruments limits the availability of these networks. Ambient Air Quality Directive 2008/50/EC of the European Union proposes two different uncertainty levels for PM monitoring devices; 25% for continuous and 50% for indicative monitoring devices [1]. According to this directive, it is worth considering if PM concentrations could be measured with cheaper and more versatile methods, which would still adhere to the indicative measurement uncertainty limit. This would allow for the densification of existing networks and therefore improve their coverage [2,3,4,5].

Several inexpensive aerosol sensors are currently available on the markets [6,7,8,9]. These sensors typically operate under the principle of light scattering detection; a light source (infrared LED or laser) is positioned in an angle with respect to a photodetector. Particles passing through the light beam illuminate scattered light, which is detected by the photodetector. The generated light scattering signal is filtered and amplified by the electronics, and an analogue voltage or pulse width modulation is used to interpret the measured conditions. This simple design can be achieved with a relatively few number of parts, which presumably is the main reason for the inexpensiveness. Additionally, resulting from the simple design, majority of the aftermarket sensors have low power consumption and are handheld regarding their physical size. This makes them particularly attractive applications for monitoring networks [10]. 

However, in spite of the proposed benefits, the accuracy and precision of the inexpensive sensors remains a concern. The reference documents that are provided by the manufacturers seem to be incomplete and the user is often left unaware of the specific details of the response characteristics. The physical properties of atmospheric particles, such as size, composition, and refractive index, vary depending on the pollution source, and, furthermore, directly influence the measurements of optically operated devices [11,12]. Therefore, the specific response characteristics under different conditions have to be known in order to ensure the quality and appropriateness of the measurement data. 

Several studies have demonstrated that an adequate performance may be achieved with inexpensive sensor designs [13,14,15,16,17]. The studies have tested sensor response characteristics by producing either polydisperse test aerosols or monodisperse test aerosols of few particle sizes. Although this is being useful and a definite step forward in understanding inexpensive aerosol sensors, it leaves room for speculation whether the sensor response would behave similarly throughout the atmospheric particle size spectrum. Additionally, it proves to be difficult to differentiate sensor response between number and mass concentration if no comparisons between number concentrations, different particle sizes, and the consequent sensor responses are made.

The aim of this study was to characterize response properties of Shinyei PPD42NS and PPD60PV aerosol sensors. Characterization was done by using a novel laboratory test method, where monodisperse test aerosols of different particle sizes were continuously produced as a function of time. Simultaneously decreasing number concentration allowed for making observations whether the sensor responses followed mass or number concentration values. Furthermore, a short field test was demonstrated to highlight how the results of the laboratory tests showcased themselves in ambient conditions. For the experiments, a custom made Prototype Aerosol Sensor (PAS) was constructed. The PAS utilized both Shinyei sensors simultaneously.

## 2. Methods

### 2.1. Prototype Aerosol Sensor (PAS)

The basic designs of the Shinyei PPD60PV and PPD42NS sensors (Shinyei Technology Co., Ltd.; Kobe, Japan) are similar; an infrared LED is positioned in a forward angle with respect to a photodiode. Particles passing through the light beam scatter light, which generates a measurable signal in the sensor circuitry. The scattered light is focused on to the photodiode by a lens. While both sensors have this light scattering focusing lens, only PPD60PV has a focusing lens also for the infrared light source. According to the manufacturer, PPD60PV has a lower limit of detection of 0.5 µm. Respectively, lower limit of detection of the PPD42NS is 1 µm. This difference is likely explained by the different lens configurations. 

The measuring algorithm of the Shinyei sensors is based on a comparison of voltage levels. When the amplified photodiode signal exceeds a predefined reference voltage, a low pulse signal is emitted. The sensor output signal is then a ratio of low pulse occupancy time and total elapsed measuring time. This measuring method indicates that the Shinyei sensors are not single particle counters, since the total number of pulses is not counted, but rather photometers where the scattered light intensity is the more important measurement factor. A List of sensor properties is presented in Table 1. The used sensors were in original condition.

In general, it is common that inexpensive aerosol sensors do not feature any data acquisition hardware, and minimal effort has been devoted to the sampling configuration design. This was the case also for the Shinyei sensors. The PAS device introduced here was fabricated to address both of these issues by implementing the necessary data logging hardware along with a custom build sampling configuration. 

In the PAS application, both of the sensors were attached in series one after each other (PPD60PV first) with a steel pipe of 10 mm in diameter. Deposition losses due to the series configuration were estimated to be negligible. A schematic of the configuration is shown in Supplementary Figure S1 [18,19]. At first, the sensor modules were positioned back side first in the enclosure in order to retain the sample flow direction, which was originally designed by the manufacturer. However, after preliminary tests, it was shown that the lenses of the sensors were vulnerable to contamination, and, therefore, the orientation was decided to be reversed. This solved the problem for the most part, but consequently, the aerosol sample beam shape became suboptimal for the PPD42NS sensor. The light source was located close to the focal point of the light beam, which caused the protruded plastic frame of the LED to partly block the steel pipe that fed the sample aerosol into the detection chamber. It is likely that this caused some inertial deposition losses of larger particles. The uniqueness of the PAS sampling configuration ought to be taken into account when comparisons between different studies and varying configurations are conducted.

A tygon tube was attached between the end of the PPD42NS sensor and a miniature rotary vane vacuum pump. The sample flow was measured to be 0.9 L/min (TSI Mass flowmeter 3063). A small inline filter was added to prolong the lifespan of the pump. At the other end, a 10 mm steel pipe with a regular 10 mm pipe fitting was added. The pipe fitting was connected to union tee connector, which was used as an inlet of the enclosure. Union tee fitting also enabled sample temperature and humidity monitoring. A Sensirion SHT71 sensor was used to measure the sample condition and an additional TMP36GZ analog sensor was used to measure the ambient temperature inside the enclosure.

Sensor modules were housed in an IP65 proof casted aluminium alloy case. The case dimensions were 200 × 125 × 75 mm, and it weighed 940 grams (other parts not accounted). Four LEDs were fixed at the top of the case for measured value and possible fault condition indications. The vacuum pump exhaust tube was tightly fitted to the outlet hole drilled at the bottom of the case. 

An Atmel ATmega328p chip based Arduino microcontroller unit (MCU) was used to extract the data from the sensors. Data logging shield with real time clock was used to store the data on an SD memory card with correct timestamp. The MCU had an integrated voltage regulator and it was recommended to use 7–12 V source voltage. A 7.5 V DC general purpose power supply was used to power the PAS unit. Total power consumption of the PAS was measured to be 2.25 W, where the highest power consuming part appeared to be the vacuum pump with approximately 50% share of total power consumption. The PAS unit and the sensor configuration are shown in Supplementary Figures S2 and S3.

### 2.2. Novel Laboratory Evaluation Method

The response characteristics of the PAS were evaluated in a laboratory configuration, which consisted of Vibrating Orifice Aerosol Generator (VOAG; TSI Inc., Shoreview, MN, USA) and Aerodynamic Particle Sizer 3321 (APS; TSI Inc., Shoreview, MN, USA). The VOAG was used to generate various sample aerosols and, respectively, the APS was used as a reference measurement instrument. For the VOAG, an additional syringe pump with an external syringe feeder was added in parallel with the existing syringe pump setup. The separate syringe pumps were connected to the VOAG solution feed line with a manually operated three-way valve. This allowed for the switching of solution feeds between different syringe pumps uninterruptedly. Both the VOAG and APS instruments were otherwise in standard condition.

**Figure 1:**
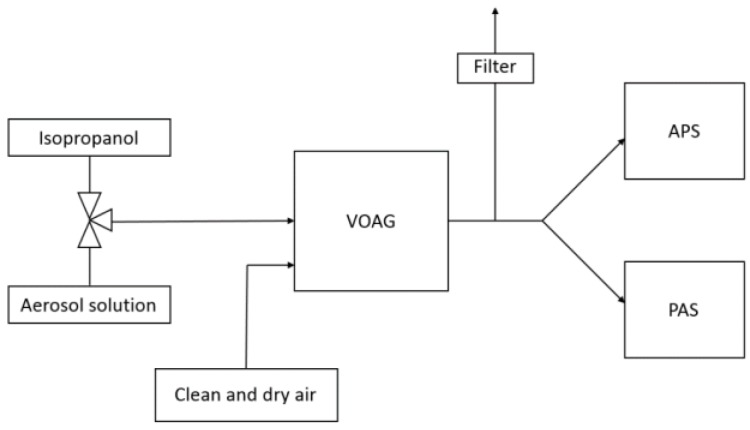
The sampling configuration used in the laboratory experiment. Manually operated three-way valve controlled the isopropanol and aerosol solution feeds.

Sample aerosols generated by the VOAG were fed through a sampling line of 25 mm in diameter to a flow splitting section which divided the sample aerosol to APS and PAS symmetrically. A schematic of the configuration is presented in Figure 1. A filtered exhaust was used to prevent over pressurization of the system due to high flow rate of dilution air.

The VOAG was operated as follows: an isopropanol solvent was first fed through the system to generate small particles. These particles are formed due to the nonvolatile impurities within the solvent. After a stable aerosol generation was achieved, the isopropanol solvent was switched to a high concentration aerosol solution. The switch was carried out uninterruptedly by using the three-way valve. No operating parameters were changed. This technique results in a gradient-like generation of different size monodisperse aerosols as a function of time, and, hence, eliminates the need to separately mix solutions of varying concentrations. The amount of work is greatly reduced and detailed observations can be made about the correlations between sensor response and particle size. A single test run lasts less than 15 min. 

Due to the constant output of the VOAG, this technique is particularly useful when differentiating the response between particle number and mass concentration. At first, the number of total particles that are measured is high. Once the particle size starts to increase, the naturally occurring inertial deposition losses also increase, and thus the overall measured number concentration starts to decrease. However, simultaneously the total mass concentration increases since the particle mass is proportional to the cube of the particle diameter. Therefore, it is easy to make observations whether the sensor response start to follow the mass or the number concentration curve. 

The limiting feature of this test method is the inability to produce particles smaller than the evaporation residual of the isopropanol solvent allows. Furthermore, the uncertainty of the APS counting efficiency increases gradually when submicron particles are being measured.

Two different substances were used separately in the aerosol solutions; dioctyl sebacate (DOS) and palmitic acid. DOS is a liquid oil (ρ = 0.914 g/cm^3^), which generates transparent particles. Palmitic acid generates white colored crystalline particles (ρ = 0.85 g/cm^3^). The two different substances allowed for making comparisons about whether these differences in particle composition had any effect on the sensor response. The different densities of the two particle types were accounted, when the aerodynamic diameters were converted into physical diameters. The sampling resolution of both APS and PAS was 10 s. The parameters that were used in the VOAG are listed in Table 2.

### 2.3. Field Validation

#### 2.3.1. Reference Instrument and Configuration

A model 1.108 GRIMM Aerosol Spectrometer (0.23–20 µm with 15 channels) was used as a reference instrument in the field validation. The GRIMM is a medium cost ambient air monitoring instrument with detection properties that are similar to the APS [20]. Furthermore, the GRIMM detects particles optically, as does the PAS. The more sophisticated GRIMM was therefore considered to be a good reference point when evaluating the performance of a much cheaper, optically operated aerosol sensor. The previous factory calibration of the GRIMM was valid during the experiment.

Both GRIMM and PAS units were housed inside of a container, which had heating and electricity installed. A 25 mm diameter sample line was taken through the ceiling, and a standard-like PM_10_ inlet was used as a sampling inlet. Inside of the container, the vertical sample line was split to the GRIMM and PAS units separately. Sampling flow rate of the GRIMM was 1.2 L/min. Deposition losses were calculated to be negligible.

#### 2.3.2. Measurement Site

The field measurement was conducted at Station for Measuring Ecosystem—Atmosphere Relationships (SMEAR III) monitoring station (60°12′ N, 24°58′ E, 26 m above sea level) in Helsinki. Helsinki, alongside with Espoo, Kauniainen, and Vantaa, forms the Helsinki metropolitan area with more than a million inhabitants. Helsinki metropolitan area is located on a coastal area by the Baltic Sea, which separates the metropolitan area from the European mainland. 

The surrounding area of SMEAR III station is rather heterogeneous consisting of patched forest and low vegetation, buildings, parking lots, and roads [21]. The closest main road, which leads to Helsinki City center, is located approximately 200 m away in southeast direction with a traffic rate of 47,000 vehicles per workday [22].

Particulate matter concentrations are typically much lower in Finland than in other European countries [22,23,24,25]. Significantly elevated particle matter concentration time periods (episodes) are usually resulted from long-range transported pollution originating from eastern and central European countries or from local street dust resulting from sanded icy roads and winter tires [26,27,28,29]. Local fine particulate matter sources in Helsinki area are traffic, wood combustion, and secondary aerosol formation from biogenic and anthropogenic precursor gases [30,31,32].

## 3. Results and Discussion

### 3.1. Laboratory Test Results

Both of the sensors were first tested for measurement bias, and it was discovered that the PPD60PV sensor on average yielded a value of 0.62 when clean air was fed through the system. This bias value was subtracted from the measured values when data analysis was performed. The bias for the PPD42NS was zero. 

The detection efficiency of the APS proved to be uncertain for particles that were smaller than approximately 0.8 µm. This was confirmed when comparisons between total particle counts of the GRIMM and APS were made in collateral laboratory tests. The tests also showed that the GRIMM seemed to classify the sizes of small particles differently than the APS. For example, when particles of pure isopropanol were produced, the GRIMM estimated the particle size to be within 0.23–0.4 µm, whereas the APS classified the particles to be approximately 0.6 µm in size. Since the calculated theoretical particle size was 0.5 µm, and, it was assumed that the APS was more trustworthy regarding size classification of particles. 

#### 3.1.1. Transparent Liquid Dioctyl Sebacate (DOS)

**Figure 2:**
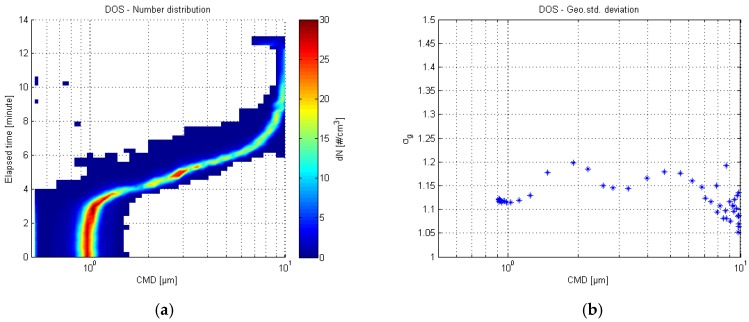
Aerodynamic Particle Sizer (APS) number distribution of the dioctyl sebacate (DOS) test (**a**) and the respective geometric standard deviation plot (**b**).

The left surface plot of the Figure 2 presents the number distribution of the APS. The color indicates the concentration level. In the right, the geometric standard deviation ($\sigma_{g}$) is shown as a function of count median diameter (CMD). Mass median diameter (MMD) values were approximately the same as CMD values. The test lasted for 13 min, and the essential size distribution shift for four minutes. The geometric standard deviation values were under 1.25, which is in an indication that the sample aerosol was monodisperse.

**Figure 3:**
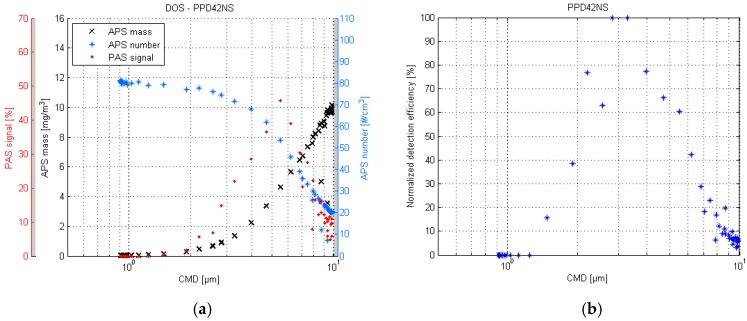
PPD42NS response plot of the DOS test (**a**) and the respective normalized detection efficiency scatter plot (**b**).

The DOS response curve of the PPD42NS is shown in the left of Figure 3. The previously described phenomenon (see Section 2.2) of particle deposition losses can be identified in the figure, and the sensor response seems to follow more closely to the mass values rather than number concentration. The sensor signal peaks at 5.8 µm and gradually diminishes in the larger particle sizes. 

On the right of the Figure 3, the detection efficiency of the PPD42NS is illustrated as a normalized ratio of PAS signal and APS mass. It appears that the highest detection efficiency is achieved in particle size range 2.5–3.5 µm. The lower limit of detection appears to be approximately 1.3 µm. The sensor response for particles larger than 6 µm is as weak as the response for particles that are smaller than 2 µm.

**Figure 4:**
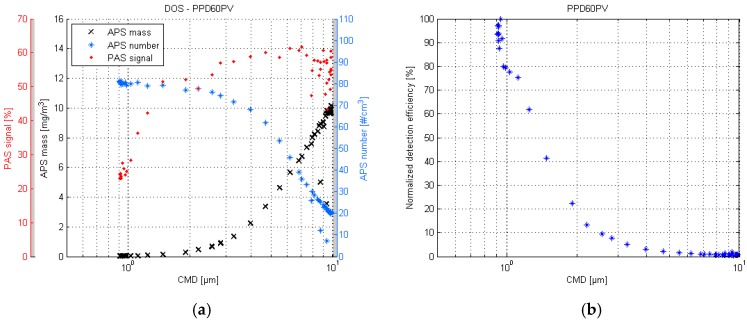
PPD60PV response plot of the DOS test (**a**) and the respective normalized detection efficiency scatter plot (**b**).

The respective PPD60PV response plots are shown in Figure 4. Similarly to the PPD42NS, the PPD60PV seems to follow the mass concentration values. The highest detection efficiency is achieved in particle sizes of below 1 µm. The response for particles larger than 2 µm is practically zero.

The difference in PPD42NS and PPD60PV response plots is unexpected in sense that the saturation, which occurs for the PPD60PV, cannot be observed for the PPD42NS despite the same measuring techniques. In this case it is likely that the previously mentioned suboptimal aerosol sample beam shape of the PPD42NS sensor caused the diminishing response for larger particles. The sample aerosol had to make a slight curve before entering into the detection volume, and therefore some of the larger particles probably impacted onto the sidewall.

#### 3.1.2. White Crystalline Palmitic Acid

**Figure 5:**
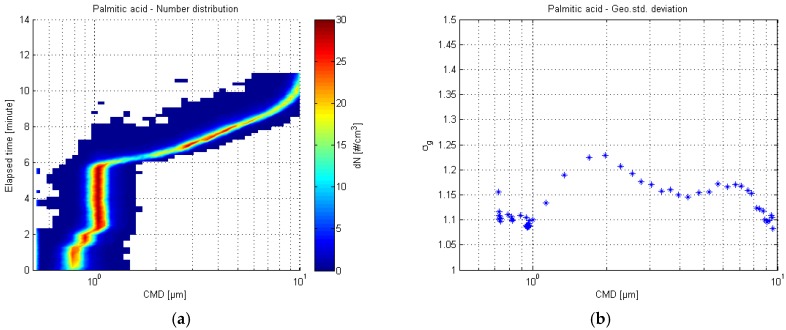
APS number distribution of the palmitic acid test (**a**) and the respective geometric standard deviation plot (**b**).

Particle number distribution of the palmitic acid test run and the respective geometric standard deviation plot are shown in Figure 5. The sample aerosol was monodisperse, and the test lasted for 11 min. The inconsistencies at the beginning of the test resulted from pressure differences between aerosol and isopropanol syringes. The aerosol syringe had to be pre-pressurized before the syringe switch, since otherwise the VOAG aerosol jet would have stopped due to the sudden loss of liquid pressure. The pre-pressurization in this case lead to slight overpressure, which in turn directly translated into higher solution feed rate and larger particles. The aerosol generation remained constant after the liquid pressure had stabilized. 

**Figure 6:**
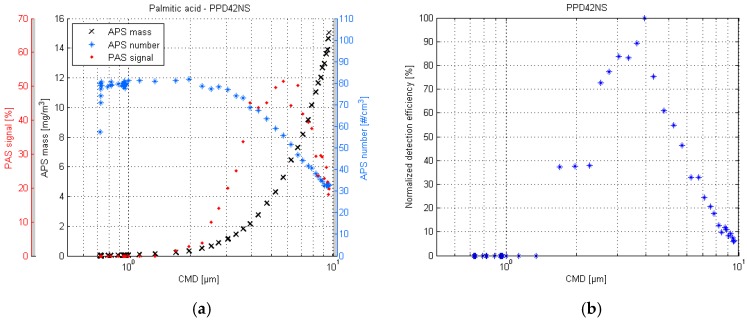
PPD42NS response plot of the palmitic acid test (**a**) and the respective normalized detection efficiency scatter plot (**b**).

The smallest generated particle size in this test was smaller than in DOS test, approximately 0.72 µm. From the Figure 6 it can be observed that the APS detection efficiency is starting to destabilize. Regarding the smallest particles, it appears that in this sensor characterization setup, the limiting factor is the APS detection efficiency, rather than the VOAG output capability.

For the PPD42NS, the response curve shape is similar to the DOS response, as can be seen in Figure 6. The highest detection efficiency is in the particle size range of 3–4 µm, and the lower limit of detection is approximately 1.5 µm. The slight shift in peak detection efficiency could be explained by the bouncing effect of the crystalline palmitic acid particles. Particle bounce is an inherit property of solid particles, in particular, where the particle bounces off of a supposed impaction surface instead of adhering to it, as liquid particles tend to do [33]. In this case, the bouncing would lead to better penetration of larger particles, and, therefore, would also support the suspicion that the reason for weak large particle detection is the lack of particles present due to impaction rather than the inability of the sensor to measure them. The better penetration efficiency can also be seen in the APS measurements, which indicates that the deposition losses are smaller also in the common sampling lines.

**Figure 7:**
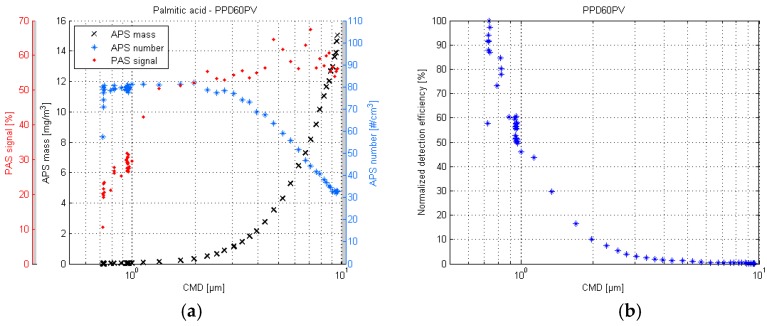
PPD60PV response plot of the palmitic acid test (**a**) and the respective normalized detection efficiency scatter plot (**b**).

The palmitic acid responses of the PPD60PV are shown in Figure 7. The response seems to be similar to the DOS response. The highest detection efficiency is achieved in particle sizes below 1 µm, and the signal diminishes quickly in particles larger than 1 µm.

**Figure 8:**
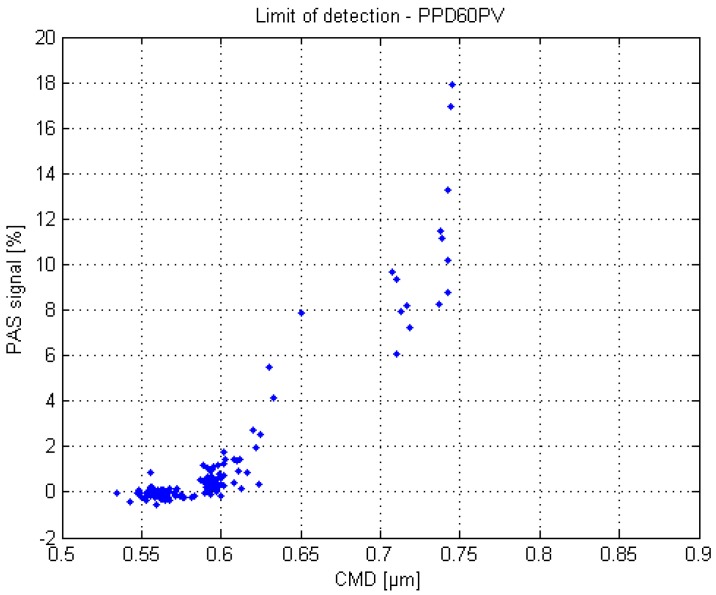
Estimated lower limit of detection of the PPD60PV sensor. Particles were produced with isopropanol by altering the liquid pressure.

The lower detection limit of the PPD60PV is presented in Figure 8. As in the previous tests, the aerosol was monodisperse and the generated particle number concentration remained the same, since the droplet breakup frequency and air flow rate (dispersion and dilution) were kept constant. The different particle sizes were produced with pure isopropanol by altering the liquid pressure. The assumption was that the APS was still able to classify the particle sizes correctly, even though the measured number and mass concentrations were misleading due to the inefficient counting. The correctness of the APS particle size classification was supported by theoretical calculations, which indicated that the minimum particle size in this test would be approximately 0.5 µm.

The Figure 8 indicates that the lower detection limit of the PPD60PV is approximately 0.55 µm. The negative values are resulting from the subtraction of the average bias value. When compared to the Figure 7, it appears that the sensor response collapses soon after 0.75 µm, and quickly reaches zero before 0.5 µm. It seems that the optimal detection range of the PPD60PV is quite narrow, approximately 0.7–1 µm.

### 3.2. Field Validation Results

The field experiment with PAS sensor was carried out in February from 1st till 18th of February. A total of 408 h average data points were recorded. During the measurement campaign, some long range fine particle transport episodes (2–5, 11, and 17 February) and local coarse mode episodes (7–10 and 15 February) were recorded. The measurement campaign time period represented quite typical meteorological conditions regarding temperature and relative humidity for that time of the year [34]. The temperature and relative humidity observations are presented in Supplementary Figure S4. The depth of the snow cover was about 7 cm throughout the experiment.

**Figure 9:**
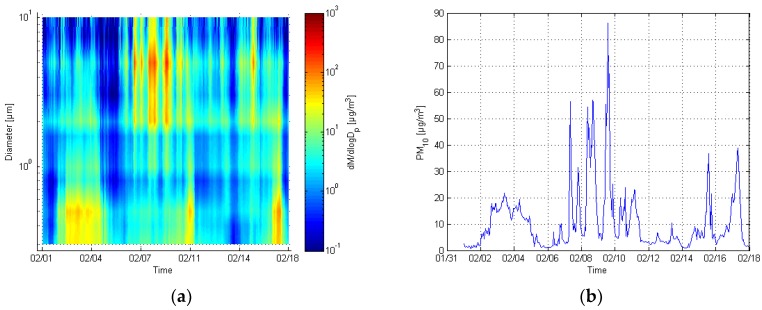
Normalized mass distribution of the GRIMM (**a**) and respective PM_10_ values (**b**).

A normalized size distribution of particle mass concentration and total particle mass values of the GRIMM are presented in Figure 9. Mass concentration unit was used to represent the results, because of its apparent correlation with the sensor responses. The different episodes, which occurred during the experiment can be identified in the figure. It was assumed that the coarse mode episodes were probably caused by the dry weather, which promoted street dust generation. Fine mode episodes were most likely caused by long range transportations of fine particles.

**Figure 10:**
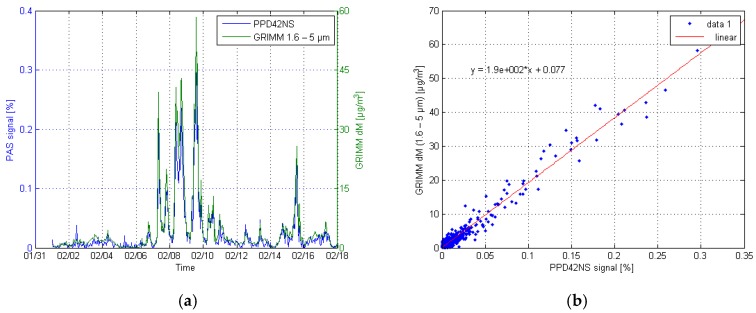
Time series (**a**) and correlation plot (**b**) of the PPD42NS and GRIMM channels 8–11.

**Figure 11:**
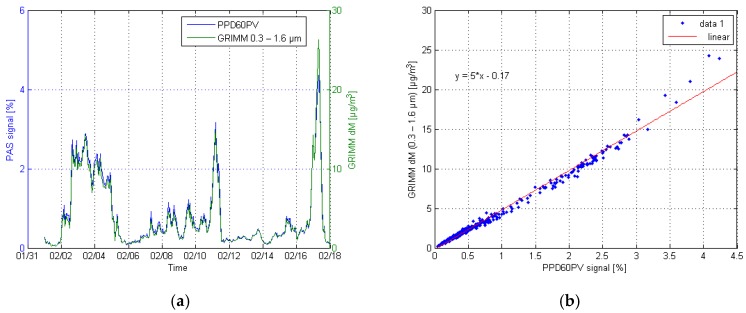
Time series (**a**) and correlation plot (**b**) of the PPD60PV and GRIMM channels 2–7.

Correlation and time series plots of both Shinyei sensors and GRIMM are shown in Figure 10 and Figure 11. The reference data of the GRIMM was chosen in a way that the best correlation could be achieved. This meant that the representative particle size channels of the GRIMM were manually chosen, and then compared one by one to the sensor responses. For the PPD42NS, the best correlation (R^2^ = 0.962) was found for channels 8–11, which marks for the particle size range of 1.6–5 µm. For the PPD60PV, the respective channels were 2–7 (R^2^ = 0.986) and the consequent particle sizes 0.3–1.6 µm.

The field test results are in line with the observations that were made in the laboratory experiments. The only difference appears to be the lower limit detection of the PPD60PV; the GRIMM estimates it to be 0.3 µm, whereas the APS indicated a value of 0.55 µm. When considering the previously mentioned differences in particle size classification of GRIMM and APS (see Section 3.1), it appears that the difference apply also in this case and is therefore consistent.

According to our laboratory and field experiment results, it is evident that varying sensor performances are expected if standard PM fractions of 2.5 and 10 µm are used as a reference values. Neither of the sensors captures the standard PM_2.5_ or PM_10_ fraction completely, and, therefore, the continuously changing size distribution will inevitably lead to mixed results. Correlation may be high or low, depending on how the present size distribution compliments the specific detection efficiency characteristics of the given sensor. However, the size distribution of atmospheric particles is usually bimodal. Particularly in this case, the majority of the mass is located in particles that are smaller than 1 µm and are larger than 2 µm. Additionally, the bimodal distributions tend to be wider than the viable detection efficiency ranges of the sensors. When considering this, it is probable that the Shinyei sensors could measure the PM_2.5_ and PM_10-2.5_ size fractions with indicatively valid accuracy.

## 4. Conclusions

The experiments conducted in this study showed that both Shinyei sensors respond to mass concentration stimulus instead of number concentration. Particle size seemed to be the biggest factor that was affecting the sensor response; the most efficient detection size range for the PPD42NS was approximately 2.5–4 µm. Respectively, the most efficient detection range for the PPD60PV was approximately 0.7–1 µm. Particle composition seemed to have a minor effect on the PPD42NS response. Lower limit of detection of both sensors seemed to be close, although optimistic, to the ones declared by the manufacturer (1 µm and 0.5 µm). Both of the sensors exhibited high correlation (PPD42NS: R^2^ = 0.962, PPD60PV: R^2^ = 0.986) for the valid detection ranges of 1.6–5 µm and 0.3–1.6 µm, when field measurement comparisons to a medium price optical dust monitor were conducted. According to these results, neither of the sensors would benefit of size-selective inlets due to the inherit property of response amplification of only certain particle sizes. Due to the bimodal distribution of atmospheric particles, it is likely that the PPD42NS sensor could be used to indicatively measure PM_10–2.5_ size fraction. Similarly, the PPD60PV could possibly be used to measure PM_2.5_ size fraction. Internal precision of the PAS was not tested in the experiments.

Understanding of sensor specific response characteristic is a prerequisite to the reliable interpretation of measurement data. We note that the user of inexpensive aerosol sensors is required to acknowledge the sensor specific response characteristics, and, furthermore, the requirements set by the measured aerosol type are related to the given measurement site environment. Having appropriate experience in operating and maintaining sensors is also advisable. If the sensor data quality is proven to adhere to a given criteria, inexpensive aerosol sensors could be used to complement existing air quality measurement networks by increasing the spatiotemporal coverage. The increased coverage would benefit air quality models and predictions, and therefore improvements in public health could be achieved.

## Figures and Tables

**Table 1:** List of properties of the Shinyei sensors declared by the manufacturer.

Model	PPD42NS	PPD60PV
Origin	Shinyei Technology Co., Ltd., Kobe, Japan	Shinyei Technology Co., Ltd., Kobe, Japan
Dimensions (mm)	59 × 42 × 22	88 × 60 × 22
Weight (g)	24	36
Power consumption (W)	0.45	0.7
Supply voltage (VDC)	5	5
Particle size detection (µm)	>1.0	>0.5
Wavelength (nm)	940	940
Type (-)	Forward angle, photometer	Forward angle, photometer
Output signal	Pulse width modulation	Pulse width modulation
Operating temperature (°C)	0–45	0–45
Operating humidity (%)	<95%	<95%
Price (€)	~70	~150

**Table 2:** The Vibrating Orifice Aerosol Generator (VOAG) parameters.

Parameter	Value
Dilution air flow rate	20 L/min
Dispersion air flow rate	1500 cc/min
Liquid feed rate	~2.98 mm^3^/s
Droplet breakup frequency	45 kHz
VOAG orifice diameter	20 µm
DOS solution	10 g/L 2-propanol
Palmitic acid solution	10 g/L 2-propanol
2-propanol	Max. evaporation residual 0.0001%

## Supplementary Materials

The following are available online at http://www.mdpi.com/1424-8220/17/12/2915/s1, Figure S1: A Schematic of the sensor configuration, Figure S2: The PAS unit, Figure S3: A Schematic of the PAS, Figure S4: Meteorological conditions during the field experiment.

## References

[1] Official Journal of the European Union (2008). Directive 2008/50/EC of the European Parliament and the Council of 21 May 2008 on Ambient Air Quality and Cleaner Air for Europe.

[2] Heimann I., Bright V.B., McLeod M.W., Mead M.I., Popoola O.A.M., Stewart G.B., Jones R.L. (2015). Source Attribution of Air Pollution by Spatial Scale Separation using High Spatial Density Networks of Low Cost Air Quality Sensors. Atmos. Environ..

[3] Moltchanov S., Levy I., Etzion Y., Lerner U., Broday D.M., Fishbain B. (2015). On the Feasibility of Measuring Urban Air Pollution by Wireless Distributed Sensor Networks. Sci. Total Environ..

[4] Rajasegarar S., Zhang P., Zhou Y., Karunasekera S., Leckie C., Palaniswami M. High Resolution Spatio-Temporal Monitoring of Air Pollutants using Wireless Sensor Networks. Proceedings of the 2014 IEEE Ninth International Conference on Intelligent Sensors, Sensor Networks and Information Processing (ISSNIP).

[5] Jerrett M., Donaire-Gonzalez D., Popoola O., Jones R., Cohen R.C., Almanza E., de Nazelle A., Mead I., Carrasco-Turigas G., Cole-Hunter T. (2017). Validating Novel Air Pollution Sensors to Improve Exposure Estimates for Epidemiological Analyses and Citizen Science. Environ. Res..

[6] Holstius D.M., Pillarisetti A., Smith K.R., Seto E. (2014). Field Calibrations of a Low-Cost Aerosol Sensor at a Regulatory Monitoring Site in California. Atmos. Meas. Tech..

[7] Jiao W., Hagler G., Williams R., Sharpe R., Brown R., Garver D., Judge R., Caudill M., Rickard J., Davis M. (2016). Community Air Sensor Network (CAIRSENSE) Project: Evaluation of Low-Cost Sensor Performance in a Suburban Environment in the Southeastern United States. Atmos. Meas. Tech..

[8] Gao M., Cao J., Seto E. (2015). A Distributed Network of Low-Cost Continuous Reading Sensors to Measure Spatiotemporal Variations of PM2.5 in Xi’an, China. Environ. Pollut..

[9] Rai A.C., Kumar P., Pilla F., Skouloudis A.N., Di Sabatino S., Ratti C., Yasar A., Rickerby D. (2017). End-User Perspective of Low-Cost Sensors for Outdoor Air Pollution Monitoring. Sci. Total Environ..

[10] Kumar P., Morawska L., Martani C., Biskos G., Neophytou M., Di Sabatino S., Bell M., Norford L., Britter R. (2015). The Rise of Low-Cost Sensing for Managing Air Pollution in Cities. Environ. Int..

[11] Baron P.A., Willeke K. (2001). Aerosol Measurement: Principles, Techniques, and Applications.

[12] Hinds W.C. (1999). Aerosol Technology: Properties, Behaviour, and Measurement of Airborne Particles.

[13] Kelly K.E., Whitaker J., Petty A., Widmer C., Dybwad A., Sleeth D., Martin R., Butterfield A. (2017). Ambient and Laboratory Evaluation of a Low-Cost Particulate Matter Sensor. Environ. Pollut..

[14] Wang Y., Li J., Jing H., Zhang Q., Jiang J., Biswas P. (2015). Laboratory Evaluation and Calibration of Three Low-Cost Particle Sensors for Particulate Matter Measurement. Aerosol Sci. Technol..

[15] Sousan S., Koehler K., Thomas G., Park J.H., Hillman M., Halterman A., Peters T.M. (2016). Inter-Comparison of Low-Cost Sensors for Measuring the Mass Concentration of Occupational Aerosols. Aerosol Sci. Technol..

[16] Sousan S., Koehler K., Hallett L., Peters T.M. (2016). Evaluation of the Alphasense Optical Particle Counter (OPC-N2) and the Grimm Portable Aerosol Spectrometer (PAS-1.108). Aerosol Sci. Technol..

[17] Zikova N., Hopke P.K., Ferro A.R. (2017). Evaluation of New Low-Cost Particle Monitors for PM2.5 Concentrations Measurements. J. Aerosol Sci..

[18] Shinyei Technology Co., Ltd. (2010). Technical Specifications Sheet: PPD42NS.

[19] Shinyei Technology Co., Ltd. (2010). Technicial Specifications Sheet: PPD60PV.

[20] Peters T.M. (2006). Comparison of the Grimm 1.108 and 1.109 Portable Aerosol Spectrometer to the TSI 3321 Aerodynamic Particle Sizer for Dry Particles. Ann. Occup. Hyg..

[21] Vesala T., Järvi L., Launiainen S., Sogachev A., Rannik U., Mammarella I., Siivola E., Keronen P., Rinne J., Riikonen A. (2008). Surface-Atmosphere Interactions Over Complex Urban Terrain in Helsinki, Finland. Tellus Ser. B Chem. Phys. Meteorol..

[22] Järvi L., Rannik U., Mammarella I., Sogachev A., Aalto P.P., Keronen P., Siivola E., Kulmala M., Vesala T. (2009). Annual Particle Flux Observations Over a Heterogeneous Urban Area. Atmos. Chem. Phys..

[23] Timonen H., Aurela M., Carbone S., Saarnio K., Frey A., Saarikoski S., Teinilä K., Kulmala M., Hillamo R. (2014). Seasonal and Diurnal Changes in Inorganic Ions, Carbonaceous Matter and Mass in Ambient Aerosol Particles at an Urban, Background Area. Boreal Environ. Res..

[24] Sillanpää M., Hillamo R., Saarikoski S., Frey A., Pennanen A., Makkonen U., Spolnik Z., Van Grieken R., Branis M., Brunekreef B. (2006). Chemical Composition and Mass Closure of Particulate Matter at Six Urban Sites in Europe. Atmos. Environ..

[25] Sillanpää M., Frey A., Hillamo R., Pennanen A.S., Salonen R.O. (2005). Organic, Elemental and Inorganic Carbon in Particulate Matter of Six Urban Environments in Europe. Atmos. Chem. Phys..

[26] Sillanpää M., Saarikoski S., Hillamo R., Pennanen A., Makkonen U., Spolnik Z., Van Grieken R., Koskentalo T., Salonen R.O. (2005). Chemical Composition, Mass Size Distribution and Source Analysis of Long-Range Transported Wildfire Smokes in Helsinki. Sci. Total Environ..

[27] Niemi J.V., Tervahattu H., Vehkamäki H., Martikanen J., Laakso L., Kulmala M., Aarnio P., Koskentalo T., Sillanpää M., Makkonen U. (2005). Characterization of Aerosol Particle Episodes in Finland Caused by Wildfires in Eastern Europe. Atmos. Chem. Phys..

[28] Karppinen A., Härkönen J., Kukkonen J., Aarnio P., Koskentalo T. (2004). Statistical Model for Assessing the Portion of Fine Particulate Matter Transported Regionally and Long Range to Urban Air. Scand. J. Work Environ. Health.

[29] Saarikoski S., Sillanpää M., Sofiev M., Timonen H., Saarnio K., Teinilä K., Karppinen A., Kukkonen J., Hillamo R. (2007). Chemical Composition of Aerosols during a Major Biomass Burning Episode Over Northern Europe in Spring 2006: Experimental and Modelling Assessments. Atmos. Environ..

[30] Timonen H., Saarikoski S., Tolonen-Kivimäki O., Aurela M., Saarnio K., Petäjä T., Aalto P.P., Kulmala M., Pakkanen T., Hillamo R. (2008). Size Distributions, Sources and Source Areas of Water-Soluble Organic Carbon in Urban Background Air. Atmos. Chem. Phys..

[31] Saarnio K., Teinilä K., Aurela M., Timonen H., Hillamo R. (2010). High-Performance Anion-Exchange Chromatography-Mass Spectrometry Method for Determination of Levoglucosan, Mannosan, and Galactosan in Atmospheric Fine Particulate Matter. Anal. Bioanal. Chem..

[32] Saarikoski S., Timonen H., Saarnio K., Aurela M., Järvi L., Keronen P., Kerminen V., Hillamo R. (2008). Sources of Organic Carbon in Fine Particulate Matter in Northern European Urban Air. Atmos. Chem. Phys..

[33] Dzubay T.G., Hines L.E., Stevens R.K. (1976). Particle Bounce Errors in Cascade Impactors. Atmos. Environ..

[34] Pirinen P., Simola H., Aalto J., Kaukoranta J., Karlsson P., Ruuhela R. (2012). Tilastoja Suomen Ilmastosta 1981–2010.

