# Supplementary figures and images for: Response Characterization of an Inexpensive Aerosol Sensor
Source: Sensors (Basel). 2017 Dec 15;17(12):2915. doi: 10.3390/s17122915 (PMC5751569; doi:10.3390/s17122915)

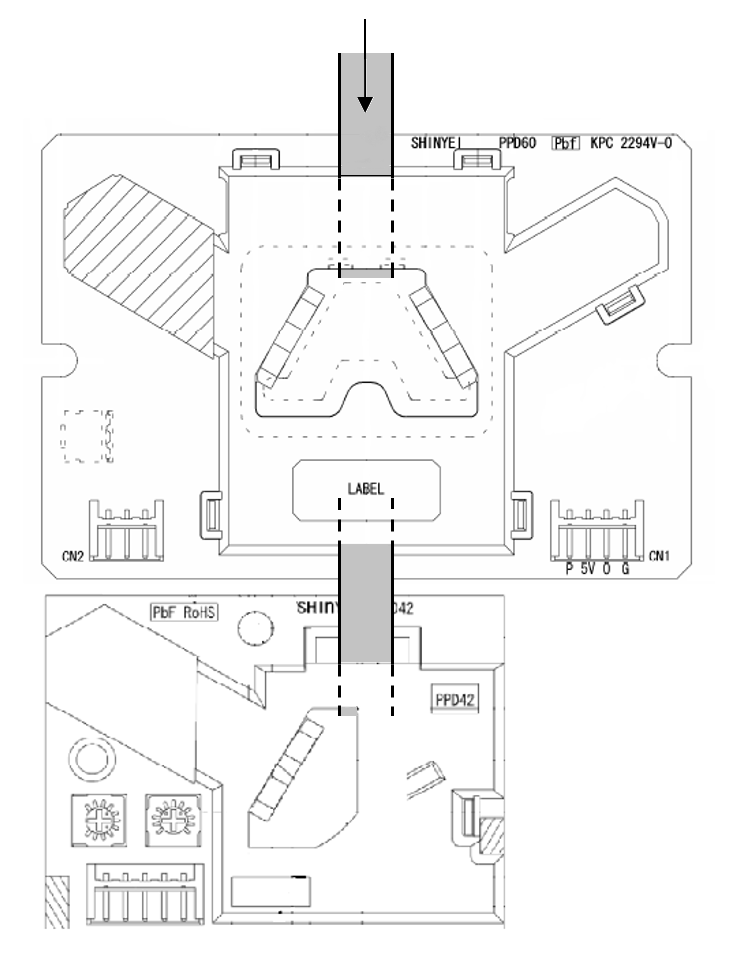

Supplement: Supplementary file 1 [file sensors-17-02915-s001.zip › Response characterization of an inexpensive aerosol sensor - supplementary figures/FigureS1-A Schematic of the sensor configuration.png]

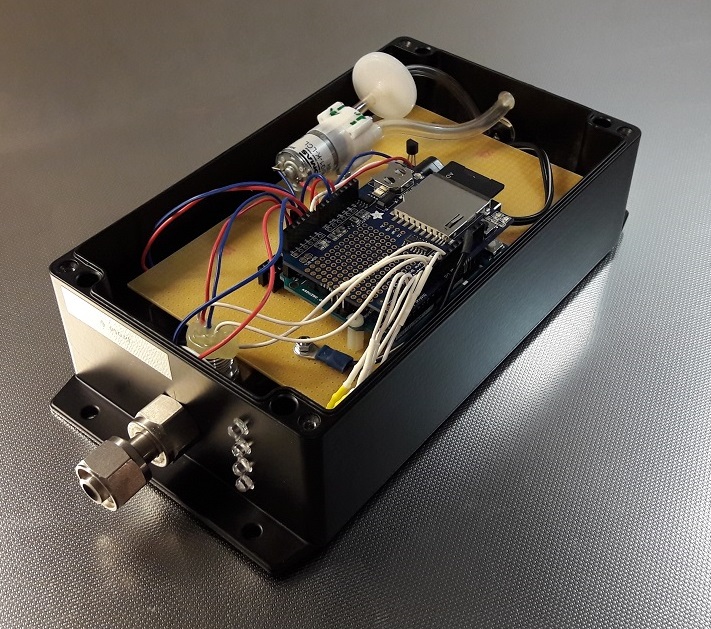

Supplement: Supplementary file 1 [file sensors-17-02915-s001.zip › Response characterization of an inexpensive aerosol sensor - supplementary figures/FigureS2-The Pas unit.png]

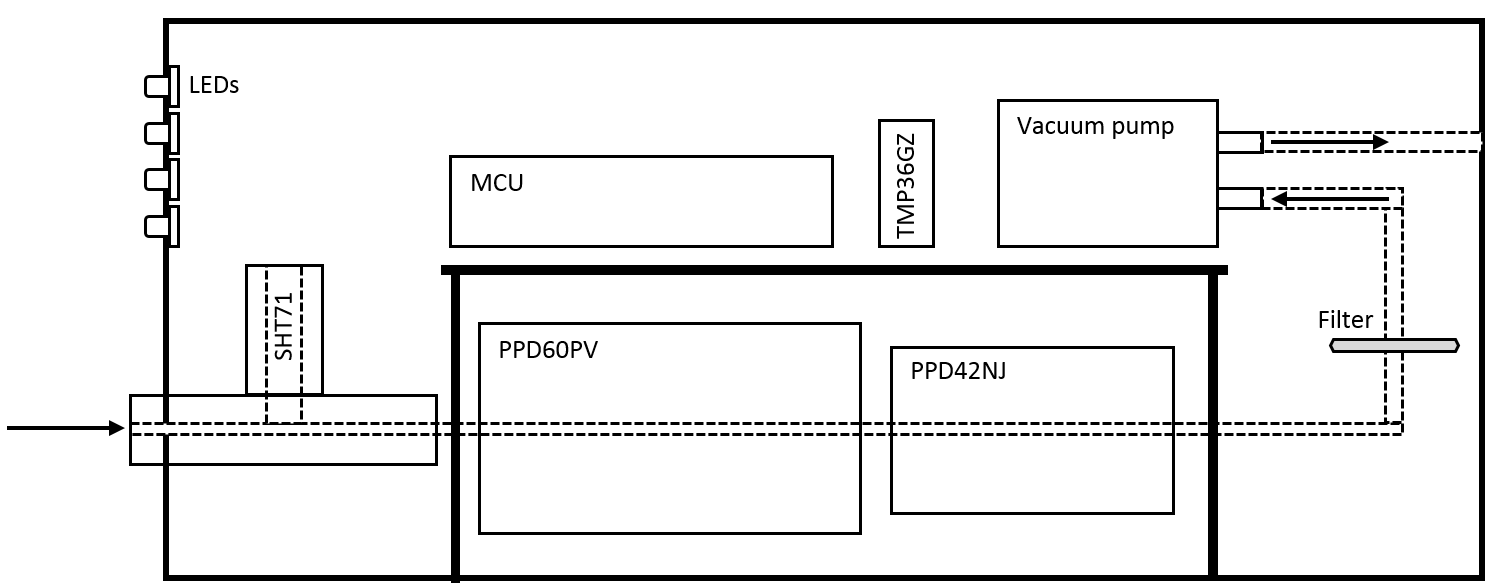

Supplement: Supplementary file 1 [file sensors-17-02915-s001.zip › Response characterization of an inexpensive aerosol sensor - supplementary figures/FigureS3-A Schematic of the PAS.png]

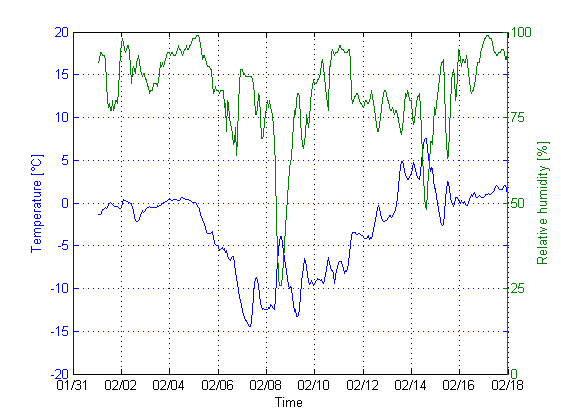

Supplement: Supplementary file 1 [file sensors-17-02915-s001.zip › Response characterization of an inexpensive aerosol sensor - supplementary figures/FigureS4-Meteorological conditions.png]
